# Genetic Dissection of Energy Deficiency in Autism Spectrum Disorder

**DOI:** 10.3390/genes16080923

**Published:** 2025-07-31

**Authors:** John Jay Gargus

**Affiliations:** 1 Medical Genetics and Genomics–Pediatrics, Physiology & Biophysics, Founding Director Center for Autism Research and Translation, University of California, Irvine, CA 92697, USA; jjgargus@neuroqure.com; 2 NeuroQure, 15375 Baranca Parkway, Suite E-104, Irvine, CA 92618, USA

**Keywords:** autism, mitochondrial disease, human accelerated regions, precuneus, diagnostics, inositol triphosphate receptor, energetics, ATP, human evolution, organelle

## Abstract

**Background/Objectives**: An important new consideration when studying autism spectrum disorder (ASD) is the bioenergetic mechanisms underlying the relatively recent rapid evolutionary expansion of the human brain, which pose fundamental risks for mitochondrial dysfunction and calcium signaling abnormalities and their potential role in ASD, as recently highlighted by insights from the BTBR mouse model of ASD. The rapid brain expansion taking place as *Homo sapiens* evolved, particularly in the parietal lobe, led to increased energy demands, making the brain vulnerable to such metabolic disruptions as are seen in ASD. **Methods**: Mitochondrial dysfunction in ASD is characterized by impaired oxidative phosphorylation, elevated lactate and alanine levels, carnitine deficiency, abnormal reactive oxygen species (ROS), and altered calcium homeostasis. These dysfunctions are primarily functional, rather than being due to mitochondrial DNA mutations. Calcium signaling plays a crucial role in neuronal ATP production, with disruptions in inositol 1,4,5-trisphosphate receptor (ITPR)-mediated endoplasmic reticulum (ER) calcium release being observed in ASD patient-derived cells. **Results**: This impaired signaling affects the ER–mitochondrial calcium axis, leading to mitochondrial energy deficiency, particularly in high-energy regions of the developing brain. The BTBR mouse model, with its unique *Itpr3* gene mutation, exhibits core autism-like behaviors and metabolic syndromes, providing valuable insights into ASD pathophysiology. **Conclusions**: Various interventions have been tested in BTBR mice, as in ASD, but none have directly targeted the *Itpr3* mutation or its calcium signaling pathway. This review presents current genetic, biochemical, and neurological findings in ASD and its model systems, highlighting the need for further research into metabolic resilience and calcium signaling as potential diagnostic and therapeutic targets for ASD.

## 1. The Rapidly Expanded Homo Sapiens Brain and ASD Vulnerability

The adult human brain accounts for only 2% of body mass but requires > 20% of the body’s basal metabolic energy, a value that approaches 60% in the neonate. Apparently, these energetic demands restricted early hominid brain volume to ~500 cc for millions of years [1,2,3], then the brain volume of early *Homo* genera slowly began to expand over the next two million years. However, about 60–70,000 years ago, the brain of early *Homo* genera underwent a rapid expansion to ~1500 cc, the current size of the modern *Homo sapiens* brain [1]. This was accompanied by a wide assortment of HAR (human accelerated region) and human-specific genetic changes that affect PUFA (polyunsaturated fatty acid) metabolism, such as the FADS (a tight linkage block of fatty acid desaturase genes, clustered at chromosome 11q12), transport (e.g., *MFSD2A*), and lipid-mediated signaling (e.g., phosphoinositides and endocannabinoids), which are all involved in shaping neuronal membranes with an influence on synaptic plasticity by the regulation of calcium signaling, and with all vulnerable to disruption in neurodevelopmental disorders [4].

Most of the Homo sapiens’ unique brain expansion occurred within the parietal lobe, particularly at the precuneus on the medial surface of the parietal lobe, just rostral to the cuneus of the occipital lobe and behind the paracentral lobule, dorsal to the splenium of the corpus callosum. This network has high synaptic densities and maintains large dendritic arbors that rely heavily on aerobic metabolism. Importantly, the precuneus is functionally connected to many regions across multiple lobes and serves as an integrative default mode network (DMN) hub that carries out visuo-spatial imagery from the first-person perspective; it is involved in self-awareness, social cognition, and memory. These are all uniquely human cognitive capacities of special interest in ASD research, as these phenotypes allude to features of ASD; this region shows both functional and structural abnormalities in ASD, including reduced connectivity and altered energy metabolism [5,6,7,8].

In ASD, mild mitochondrial dysfunction is a common finding that is evidenced by impaired oxidative phosphorylation, elevated lactate and alanine levels, carnitine deficiency, abnormal reactive oxygen species (ROS), and altered calcium homeostasis, but, much less frequently mitochondrial DNA mutations [9,10,11,12,13,14]. Recently, a large meta-analysis demonstrated that altered brain pH and lactate levels are commonly observed in many animal models of neuropsychiatric disorders and ASD, providing further evidence supporting the hypothesis that altered brain pH and lactate levels are not mere artifacts, but are rather involved in the underlying disease pathophysiology [15].

In ASD, these are primarily functional mitochondrial defects [10], and they are particularly relevant in the context of energy-hungry brain regions like the precuneus and its cortical “social brain” network, areas amongst the brain structures that display the highest resting metabolic rates [5] and vulnerabilities in ASD.

Mitochondria play a key role in energy supply to all tissues of the body, but they play a critical role in fueling the brain’s highly oxidative-dependent energy needs. The mitochondrial oxidative metabolism of glucose, the human brain’s normal energy source, provides 16 times the ATP from anaerobic metabolism. Only a few minutes of anoxia cause a rapid shutdown of the brain, leading to coma and death [16]. Even brief fasting illustrates the human brain’s high supply sensitivity due to its extraordinary energy demands. At the onset of fasting, blood glucose is quickly consumed, and the liver begins to release glucose from glycogen stores to temporarily support the brain while other tissues switch to oxidizing long-chain fatty acids. However, these fats cannot cross the blood-brain barrier (BBB), so, again, a special process is developed to support the brain when the glucose in glycogen is gone. Ketone bodies, namely, acetoacetate and β-hydroxybutyrate, which can travel on monocarboxylate transporters across the BBB, are produced for the brain by the liver to be oxidized by the mitochondria, in order to achieve a high ATP yield [17]. While other primate brains utilize ketone bodies to a limited extent, they still largely rely on glucose. The evolution of the modern human brain has not only greatly enhanced the capability of ketone body production but has also caused the generation of large fat supplies in the fetus, unique among the primates [18], to assure a secure supply of lipids for newborn brain growth and to satisfy its energy demands.

ASD may arise in part from a fundamental mismatch between the unique structural and functional expansion of more recently evolved human brain regions—particularly the association cortices involved in complex cognition—and the exceptionally high metabolic demands these regions impose. Evolutionary genomic changes, including human-specific regulatory sequences and gene networks, have reshaped certain pathways that are crucial for lipid signaling, such as the synthesis, transport, and metabolism of polyunsaturated fatty acids, which are essential for neuronal membrane integrity and synaptic function [19]. Simultaneously, these genomic shifts have introduced vulnerabilities in mitochondrial biology, affecting processes like calcium handling, oxidative phosphorylation, and reactive oxygen species detoxification. The combined effect is that newly evolved cortical circuits may operate close to metabolic limits, rendering them particularly susceptible to mitochondrial dysfunction and impaired lipid-mediated signaling, which together contribute to neurodevelopmental disruption and the diverse phenotypic spectrum observed in ASD.

Neurons make huge, fluctuating ATP demands because of variable rates of synaptic transmission and the required restorative ion pumping to re-establish the transmembrane ionic gradients of Na^+^, K^+^, and Ca^2+^. The rate of mitochondrial ATP production is homeostatically regulated to match ATP consumption in response to a signal that energy demand has increased; this signal is overwhelmingly cytosolic calcium, sensed as it is sequestered by the ER [20]. Additionally, prolonged ER calcium release can deplete ER stores, triggering store-operated calcium entry (SOCE) to replenish calcium levels and sustain mitochondrial energetics [21]. The drop in luminal ER Ca^2+^ concentration is sensed by stromal interaction molecule (STIM) proteins, especially STIM1. When ER calcium levels fall, STIM1 undergoes a conformational change and migrates to areas of the ER membrane close to the plasma membrane (ER-PM junctions). STIM1 physically interacts with Orai1, a calcium channel in the plasma membrane, triggering it to open; this results in store-operated calcium entry (SOCE)—an influx of extracellular Ca^2+^ into the cytosol. SOCE replenishes ER calcium levels by allowing newly entered Ca^2+^ to be pumped back into the ER via sarcoplasmic/endoplasmic reticulum calcium ATPase (SERCA) pumps [22].

Besides serving as the major intracellular calcium store, the ER’s other major job is protein folding and post-translational modifications, which are both highly ATP-dependent and involve chaperones like BiP (GRP78), calnexin, and calreticulin. When cellular ATP levels fall, these endoplasmic reticulum (ER) energy-intensive processes of protein folding and quality control deteriorate, due to reduced SERCA pump activity. Physiologically, the pump actively transports Ca^2+^ ions from the cytosol to sequester them in the lumen of the ER, and its failure produces a drop in ER luminal Ca^2+^ that impairs proper protein folding. As a result, unfolded and misfolded proteins accumulate within the ER lumen, triggering “ER stress”. This stress, alternatively referred to as the unfolded protein response (UPR), is orchestrated by three principal ER stress sensors: IRE1, PERK, and ATF6. IRE1 (inositol-requiring enzyme 1) possesses both kinase and endoribonuclease activity; upon activation, it catalyzes the unconventional splicing of XBP1 mRNA, removing a 26-nucleotide intron and thereby producing a potent transcription factor (XBP1s) that induces the genes involved in protein folding, ER-associated degradation (ERAD), and lipid synthesis to expand their ER capacity [23]. PERK (protein kinase RNA-like ER kinase) phosphorylates the α-subunit of eukaryotic initiation factor 2 (eIF2α), globally attenuating cap-dependent protein translation to reduce the influx of new proteins into the stressed ER. However, this phosphorylation paradoxically enhances the translation of specific mRNAs, such as ATF4, a transcription factor that activates the genes related to amino acid metabolism, antioxidant responses, and apoptosis under prolonged stress [24]. ATF6 (activating transcription factor 6), meanwhile, is translocated from the ER to the Golgi upon ER stress, where it is cleaved by site-1 and site-2 proteases. The liberated cytosolic fragment (ATF6-N) migrates to the nucleus to upregulate the expression of ER chaperones (e.g., BiP/GRP78, GRP94) and ERAD components, thereby promoting the restoration of ER proteostasis [25]. Together, these pathways maintain cellular homeostasis but can trigger apoptosis if ER stress remains unresolved.

Upon stress stimulation, this physiological homeostatic regulation, mediated by the ER calcium reservoir releases calcium uniquely through its inositol 1,4,5-trisphosphate receptors (ITPRs), which are physically coupled to the mitochondrial voltage-dependent anion channel (VDAC) via the molecular chaperone GRP75 (glucose-regulated protein 75), forming an exclusive molecular bridge to the MCU (mitochondrial calcium uniporter) in the inner mitochondrial membrane; the high local calcium concentrations at mitochondrial-associated membranes (MAMs) overcome the MCU’s low Ca^2+^ affinity. Once inside the mitochondria, Ca^2+^ activates three key dehydrogenases of the tricarboxylic acid (TCA) cycle: pyruvate dehydrogenase (PDH), isocitrate dehydrogenase (IDH), and α-ketoglutarate dehydrogenase (α-KGDH). This boosts the cycle’s nicotinamide adenine dinucleotide (NADH) and flavin adenine dinucleotide (FADH_2_) production and, hence, their donation of electrons into the inner mitochondrial membrane electron transport chain (ETC), thereby enhancing ATP synthesis by oxidative phosphorylation. Hence, Ca^2+^ couples neuronal firing into a bioenergetic homeostatic loop, with a Ca^2+^-sensing arm during sequestration by the ER and an effector arm of Ca^2+^ efflux through the ER’s ITPR, coupled to the VDAC of the mitochondrion and the Ca^2+^-sensitive rate of mitochondrial ATP production [26].

Studies have demonstrated that the effector arm of this critical homeostatic loop is functionally altered in ASD. In research into fibroblasts and iPSC-derived gamma-aminobutyric acid (GABA)-ergic interneuron precursors from ASD patients, it has been demonstrated that ITPR calcium release from the ER is impaired [27,28,29,30]. Optical patch-clamp single-channel recordings reveal a molecular abnormality of ITPR gating, resulting in brief flicker openings of otherwise normal ITPR channels. These single-channel kinetic abnormalities are similar to those found in genetic seizure-, migraine-, and arrhythmia-channelopathy syndromes [31,32], suggesting that ASD is an organellar calcium channelopathy. This flicker opening is best revealed as a low flux and a slow, low calcium wave-signaling signature occurring in response to purinergic P2Y receptor-induced calcium release from the ER [28].

Our group initiated fibroblast calcium transport studies when the Autism Sequencing Consortium, along with other groups carrying out ASD resequencing studies, began being able to identify clusters of significant risk genes for ASD [33,34,35,36]. Along with clusters of transcription factors and clusters of chromatin proteins, the largest, and biophysically most tractable, clusters were synaptic calcium signaling proteins. In functionalizing this genetic clue, with plans to produce induced pleuripotent stem cell (iPSC) neurons for complete biophysical characterization, studies of the components of calcium flux in the primary skin cells themselves were undertaken and surprisingly revealed a shared defect in intracellular calcium store release in several independent skin samples of Fragile X, Tuberous Sclerosis 1, and Tuberous Sclerosis 2, all monogenic syndromes that are highly co-morbid with ASD [28]. The ryanodine receptor release channel was normal, but the component released via the ITPRs was dramatically reduced. An optical patch clamp was used to resolve the single channel kinetics of these ITPRs, and it revealed a very short open time in the monogenic mutants, with all other properties being unchanged from neurotypical controls [28]. This flicker opening suggested that the channel open state was very unstable, rapidly collapsing and thus producing a greatly reduced calcium store efflux. This could be captured in whole-cell imaging studies via P2Y receptor activation on a Molecular Devices FLIPR automated fluorescent imaging plate reader [29]. This high-throughput method was used to study not only skin samples from the monogenic ASD subjects but also subjects with typical polygenic non-syndromic ASD, documented with research-grade ADOS testing in the ASD range. Area under the curve (AUC) separation on the receiver operator characteristic (ROC) between ASD and neurotypical controls was 84%, comparable to that of the Autism Diagnostic Observation Schedule (ADOS) itself [29].

Since ITPR gating is essential for coupling calcium signaling dynamics to mitochondrial energetics through the molecular bridge between the ER ITPRs and mitochondrial VDAC pores [37], this defective gating disrupts the homeostatic energy balance axis [30]. Consequently, this results in mitochondrial energy deficiency, a vulnerability that is particularly pronounced in the developing brain, with its high energy needs. This mismatch between the evolutionary development of a complex, energy-demanding brain and its bioenergetic control could contribute to ASD susceptibility, particularly under non-optimal metabolic conditions [30]. The recent evolutionary achievement of an enlarged social brain may come with a biological cost in the form of ASD vulnerability under stressors, potentially reflecting the recent evolutionary divergence leading to modern *Homo sapiens*, a level of evolution that has not yet stabilized [38,39].

The mitochondrial energy deficiency caused by this unstable calcium gating and flux may disrupt the homeostatic energy balance; it may thereby impair developmental network maturation and contribute to social brain deficits in ASD (39). Convergent neuroimaging techniques highlight this same energy deficiency phenotype in functional brain imaging studies of ASD. Functional magnetic resonance imaging (fMRI) studies show decreased metabolic energy consumption, seen in reduced blood flow and diminished functional connectivity within the default mode network (DMN), especially in expanded areas such as the precuneus, medial prefrontal cortex, and facial recognition area of the fusiform gyrus [40,41]. Electroencephalogram (EEG) data reveal altered alpha and gamma oscillations, reflecting impaired synchronization and insufficient energy availability [42,43,44]. Most specifically, near-infrared spectroscopy (NIRS) studies show blunted mitochondrial cytochrome oxidase responses in the temporal and frontal lobes during language and social interaction tasks [45,46]. These findings suggest that ASD vulnerability and the energy-deficiency phenotype in ASD reflect compromised bioenergetic function in the newly evolved association cortices of the “social brain”, which were themselves driven by the rapidly evolving chromosomal HARs. These findings suggest that these chromosomal and brain regions are also those that are most disrupted in ASD.

## 2. A Mini-Evolutionary Case Study of the BTBR Mouse: From Abnormal Taste Preferences to Calcium Signaling-Based ASD

An evocative mini-evolutionary tale in mice tells a potent parallel story of ASD energy deficiency hiding in plain sight. The BTBR mouse is widely recognized as one of the most valuable models of typical non-syndromic autism. This inbred strain was largely developed by sib matings in the mid-20th century at Columbia and the strain was primarily maintained due to its coat color—short for Black and Tan (*a^t^/a^t^)*—and for being heterozygous for the BRrachyury T allele (*T/+*), resulting in a shortened tail, hence giving it the name BTBR [47]. Through early crosses establishing the strain in the 1950s, scientists inserted the “*tf*” tufted allele as a marker for the tightly linked lethal *T* allele, which became fixed in the colony long before its molecular identity was discovered [48]. Mary Lyon isolated the unique tufted (*tf)* locus when it appeared as a spontaneous recessive mutation that caused characteristic waves of hair loss to spread from snout to tail. The locus was mapped tightly linked to the brachyury (*T*) complex on chromosome 17 [49], and the tufted pattern of hair loss (*tf/tf*) was subsequently used as a visible marker to identify carriers of the complex lethal *T/T* locus [48,50].

The mouse *T* gene has many known mutant alleles, both spontaneous variants and lab-engineered mutations. Classic spontaneous alleles include the original mutation discovered in 1927, which causes truncated tails and missing sacral vertebrae in *T/+* heterozygotes and is embryonically lethal when homozygous (*T/T*). The recessive *t* alleles are defined by their interaction with *T* to cause tail-lessness in double heterozygotes (*T/t*) [48]. This complex locus has been intensively characterized over the years and had originally been maintained in the BTBR strain as a segregating locus, but it had been lost and was wild-type (*T+/T+*) at some time prior to the acquisition of the current strain by the Jackson Laboratory from the McArdle Laboratory in 1994; therefore, the only studies on the BTBR strain are on (*a^t^/a^t^ T+/T+ tf/tf*) [47,48,50].

BTBR mice were originally studied as models for phenylketonuria (PKU) and metabolic syndromes, showing insulin resistance, hyperinsulinemia, impaired glucose tolerance, pancreatic beta-cell dysfunction, and obesity/type 2 diabetes susceptibility [51]. They were also characterized with abnormal immune responses, being noted to have significantly higher amounts of serum IgG, IgE and of IgG anti-brain antibodies (Abs), and of IgG and IgE deposited in the brain, along with the elevated expression of cytokines, especially IL-33, IL-18, and IL-1β in the brain and an increased proportion of major histocompatibility locus (MHC) class II-expressing microglia compared to B6 mice. BTBR mice are also significantly more susceptible to listeriosis than B6 or BALB/c mice, and the Th2-like immune profile of the BTBR mice and their constitutive neuroinflammation suggests an autoimmune-like profile. The heterozygous first filial (F1) mice showed intermediate levels of Abs and cytokines [52,53,54].

Another phenotype for which the strain was studied was altered gut permeability, which was associated with microbial dysbiosis and immune dysregulation [55]. These specimens have compromised intestinal barrier function, as evidenced by increased translocation of fluorescein isothiocyanate (FITC)-dextran across the intestinal epithelium into the bloodstream, and this is accompanied by the reduced expression of tight junction proteins like occludin and zonulin-1, indicating impaired barrier integrity. They suffer dysbiosis, with the gut microbiota composition in BTBR mice differing significantly from that of control strains, and with notable alterations in bacterial genera such as *Bacteroides*, *Parabacteroides*, and *Sutterella*. These changes are associated with behavioral abnormalities and immune responses. Furthermore, the gut sustains immune activation, with the BTBR mice displaying elevated levels of pro-inflammatory cytokines, including tumor necrosis factor alpha (TNF-α) and interleukin-6 (IL-6), in colon tissues. This suggests an ongoing inflammatory response that may contribute to both GI and behavioral symptoms [55].

Compromised sensory systems were also recognized in the BTBR mice. They demonstrated distinct sensory processing anomalies such as altered nociceptive thresholds, with behavioral assessments indicating that BTBR mice have heightened sensitivity to painful stimuli, suggesting altered nociceptive processing [56].

By the early 21st century, the Monell Chemical Senses Center in Philadelphia was developing murine screens for food taste preferences [57]. Here, the BTBR strain stood out, and it was intensively studied because these animals had an unusually high taste preference for calcium solutions. Upon detailed characterization, they were also found to exhibit markedly abnormal preference scores for exemplars of the classic taste qualities of sweet, umami, and bitter, along with comparably large abnormalities in preferences for the less well-accepted taste qualities of carbohydrate, calcium, and pyrophosphate. Smaller abnormalities were present in the taste responses to sour and salty taste compounds and to two monovalent chlorides (KCl and NH_4_Cl), but not to ZnCl_2_ or the irritant capsaicin. The researchers further tried to isolate the taste preference locus but found that the same mutation is responsible for the BTBR strain’s bad hair and its odd taste preferences. This caused the researchers to focus on the “*tf*” (tufted) allele, which had arisen spontaneously and was introduced into the BTBR strain during its early history soon after 1956, all of which suggested that the signal transduction cascade in type 2 taste receptor cells was impacted by the “*tf*” allele itself [58].

Also at the turn of the 21st century, when a major component of ASD was coming to be recognized as genetic, several systematic screens of mouse strains for autism-relevant behaviors were undertaken. It was here that a strong connection between the BTBR inbred mouse strain and ASD phenotypes was discovered, and this became the phenotype for which the strain was known [50,59,60,61]. These early findings reported that among the numerous strains tested, BTBR *T+^tf^*/J mice displayed strikingly low sociability—initiating much less social interaction than typical strains—along with high levels of repetitive self-grooming. Studies explicitly framed these strain differences as “autism-like” behavioral variations.

Following up on this lead, the Crawley laboratory group at the NIMH published a detailed behavioral characterization of BTBR *T+^tf^/*J mice, confirming that the BTBR strain exhibits all three core diagnostic features of ASD: abnormal reciprocal social interactions, impaired communicative behaviors, and repetitive/stereotyped behaviors [62,63,64]. They showed, for example, that BTBR mice engage in very little social sniffing or play compared to standard C57BL/6J mice, emit fewer social vocalizations, and display repetitive grooming with higher frequency. They proposed the BTBR strain as a model of idiopathic autism, given the breadth of its behavioral phenotype. Soon after, the Jackson Laboratory and other groups embraced BTBR as the ASD model, noting the strain’s unique traits (e.g., JAX describes BTBR as exhibiting “several symptoms of autism … including reduced social interactions, unusual vocalizations, and low exploratory behavior” and a 100% incidence of corpus callosum agenesis) [65,66]. Notably, the “*T*” gene itself is not mutated in the Jackson Labs BTBR (the strain carries the wild-type *T* allele, hence, “*T+*”), so the connection to the *T* locus was historical—the BTBR name traces back to a brachyury mutation in its lineage. Nevertheless, the identification of the BTBR strain’s autism-like behavior raised the question of whether genetic factors in the *T* region (on Chr17) or elsewhere in BTBR’s genome underlie its ASD-like phenotype.

It is particularly noteworthy that while, eventually, the behavioral phenotype of BTBR helped define it as a robust non-syndromic ASD model with all three key behavioral features, all of its *other* previously discovered phenotypes, as discussed above, were never singled out as a core part of its ASD syndrome [67,68]. However, even a passing observation of the common complaints of families in an ASD clinic will call to mind the BTBR’s evocative tactile sensory issues, its unusual food sensitivity, and its GI and immune complaints, not to mention the typical chronic lactic acidosis found in many patients (and now in all 109 models of ASD from Japan [15]). While many physicians caring for patients with ASD tackle these problems, and many do consider them part of the child’s syndrome and therapy, none have yet been incorporated into the formal definition of the ASD syndrome [69].

In the meantime, the unusual taste preferences of BTBR mice drew further attention at Monell, and they carried out detailed recombination mapping of the BTBR *T+^tf^* locus [70]. Now, in the post-genome era, they have ultimately identified the *Itpr3* gene that carries the causal mutation [70]. By 2014, the JAX catalog [50] had fully described their sequencing of the mutation: “Sequencing reveals a 12 bp deletion in Exon 23 (Chr17: 27238069, Build 38.1), which codes for amino acids 983–986 of the ITPR3 type 3 IP_3_ receptor channel [70], as the mutation arose early in the history of the BTBR strain (in or soon after 1956). This mutation is not found in 18 other strains.” The JAX catalog now reports six alleles of *Itpr3* in seven different genetic backgrounds; most are constitutive or conditional knockouts, and the catalog notes that mice homozygous for a knockout allele are viable, fertile, and exhibit no apparent abnormalities in pancreatic and salivary secretion. However, only one mutation in this gene (that specific unique spontaneous “*tf*” allele in BTBR) results in the distinctive, yet poorly understood, unusual hair and the now well-understood inseparably linked low sweetness preference, as well as a full suite of model ASD phenotypes. While other alleles have been generated, there is no published report of an effort to recapitulate the now well-known “*Itpr3*^tf^” allele [50]. The region encompassing amino acids 983–986, deleted by the “tf” mutation, lies within the regulatory domain, which is crucial for modulating channel activity through interactions with various proteins and post-translational modifications. The deletion leads to a significant reduction in functional ITPR3 protein in certain tissues, such as taste receptor cells, and results in impaired taste perception. However, the mutation does not completely abolish ITPR3 function, suggesting a hypomorphic effect rather than a complete loss of function. Furthermore, this partially functional protein may confer a gain-of-function aspect to the mutation via its potential to alter the receptor’s critical regulatory mechanisms, potentially leading to aberrant calcium signaling.

With the specific identification of the mutation, the JAX catalog soon updated its BTBR designation to BTBR *T+ Itpr3^tf^*/J, and this remains the prime strain recognized for autism-like phenotypes. A large number of groups and studies continue to expand on the range of phenotypes by which BTBR mice display robust autism-like behaviors [71,72,73]: they show profoundly impaired social interactions (failing to engage in normal amicable sniffing, social play, or mating behaviors with partner mice), deficits in social communication (for instance, BTBR pups emit fewer ultrasonic distress calls, and adults have atypical vocalization patterns), and repetitive behaviors (such as excessive self-grooming and a unique vertical jumping or “inchworming” stereotypy). These behavioral traits in BTBR are so pronounced that they fulfill the mouse analogs of autism’s diagnostic triad. In fact, BTBR is often described as an idiopathic autism model with “face validity” for core autism symptoms [50,72,74].

Beyond their behavior, BTBR mice also exhibit neuroanatomical and neurodevelopmental abnormalities that are reminiscent of findings in some people with ASD. Most strikingly, BTBR mice show a 100% penetrant absence of the corpus callosum, the major tract connecting the brain’s hemispheres. This agenesis of the corpus callosum is accompanied by a markedly reduced hippocampal commissure and reduced cortical thickness in BTBR brains [65,66]. Histological studies have documented modest and selective alterations in glia, neurons, and synapses in the BTBR forebrain, along with reduced neurogenesis in the adult hippocampus. Of all the markers examined, the most distinctive changes were seen in the neurodevelopmental proteins NG2, PSA-NCAM, NeuroD, and DCX, which is consistent with aberrant development of the nervous system in BTBR mice, along with novel substrates to link callosal abnormalities and autistic behaviors [66].

MRI and histological studies have further found that BTBR brains differ in white matter and subcortical structure volumes: for example, the reduced size of the corpus callosum and internal capsule, changes in the cerebral peduncle, and volume differences in regions like the hippocampus, striatum, and globus pallidus [67]. Such changes align with the neurodevelopmental connectivity anomalies hypothesized in autism. BTBR also shows altered cortical organization—one study noted disruptions to the formation of functional cortical areas and early neurogenesis in BTBR, paralleling neurodevelopmental patterning deficits [73].

At the cellular and molecular levels, BTBR mice have been found to have neuroimmune and synaptic differences that are relevant to ASD. For instance, the BTBR mouse has an elevated baseline of activated microglia and astroglial markers in certain brain regions, and it mounts an exaggerated neuroinflammatory response compared to control mice. Induced systemic inflammation leads to significantly more microglial activation in BTBR than in B6 mice [52]. This hyper-reactivity of the immune cells in the brain may mirror the increased neuroinflammation observed in some individuals with autism.

On the synaptic side, unbiased transcriptomic and proteomic profiling of BTBR brain tissue has revealed alterations in the expression of many autism-relevant genes and proteins, characterized by multiple genetic and epigenetic aberrations [75]. While pathway analyses pointed to the disruption of several inter- and intracellular signaling pathways, many genes and proteins involved in the development and maintenance of proper connectivity within the brain were also affected. Notably, BTBR brains show dysregulated levels of BDNF (brain-derived neurotrophic factor) and SHANK3 (a synaptic scaffolding protein), among other molecules, compared to typical mice. Both BDNF and SHANK3 are critical for synaptic development and have been implicated in human ASD, so their alteration in BTBR mice provides a mechanistic clue linking BTBR strain genetics to ASD-like neural phenotypes [75]. Functionally, BTBR neurons and oligodendrocytes may develop differently—for example, previous studies have noted precocious myelination patterns in the BTBR brain (i.e., early myelin deposition in the frontal brain regions) and reduced neuronal plasticity, which could underlie some behavioral rigidities [76].

Strikingly, while the Monell group has made use of the identification of the “*tf*” locus to recognize that there is a role for the Itpr3 receptor in the signal transduction cascade in type 2 taste receptor cells through G-protein-coupled receptors (GPCR) and lipid signaling molecules [70], this mechanistic analysis has not yet been reflected in ASD studies, which continue to use increasingly broad genomic and proteomic approaches to ferret out some mechanistic connection in BTBR mice to the ASD phenotype. Despite the widespread use of BTBR mice in ASD research, no published study has yet attempted to rescue or isolate the *Itpr3*^tf^ allele using modern clustered regularly interspaced short palindromic repeats-associated endonuclease (CRISPR/Cas9) genome editing. No targeted correction or complementation cross has been reported to dissect whether Itpr3 is necessary or sufficient for the ASD-like traits in this strain [71,73].

This research gap is both a cautionary tale and an opportunity: as we increasingly use animal models to infer molecular mechanisms, we must attend closely to their full genomic context. And, in the case of BTBR, we may, unknowingly, have been using the conspicuously best choice of available in vivo models of ITPR-linked neurodevelopmental dysfunction all along. BTBR poses a unique model for translational biology in ASD. The BTBR *T+ Itpr3^tf^*/J strain, therefore, represents a natural in vivo model of altered ITPR3 function. Unlike total knockouts (0% function) or knockout heterozygotes (50% function), this hypomorphic allele apparently has some specific, unique gain-of-function characteristics, such that, uniquely, this homozygote mimics the subtle, partial ITPR-signaling deficits seen in ASD patient cells [28,29,30]. It offers a rare chance to link molecular-level dysfunction with emergent behavioral phenotypes in a widely accepted animal model.

Moreover, because BTBR mice also demonstrate immune, sensory, GI, and metabolic alterations—domains that are increasingly implicated in children’s syndromes with ASD pathophysiology—this could enable a system-level dissection of how ER ITPR calcium signaling intersects with the broader physiological domains of the “*full* ASD syndrome”.

The simplest interpretation of the aggregate data is that the *Itpr3^tf^* allele is etiologic and diagnostic of the BTBR strain, rather than simply an innocuous, linked marker to its autism-relevant behaviors. But, to date, no published experimental evidence has been attempted to link Itpr3 hypomorphic function to the BTBR neurobehavioral phenotype, and no engineered line isolates the *Itpr3^tf^* allele or rescues it to test causality. Therefore, there is a major knowledge gap in explicitly testing what seems to be the clear contribution of impaired ER calcium release via Itpr3 to ASD-like behavior in BTBR, as has been explicitly observed in ADOS-confirmed children with ASD. This most parsimonious mechanistic hypothesis appears to be one that, surprisingly, has not yet been studied. *Itpr3^tf^* is not yet considered a major ASD gene in BTBR; it is usually never mentioned in a publication and those that do will, at most, mention an effect on taste; it remains an incidental, historical marker for this strain, a strain that is best felt to typify typical polygenic nonsyndromic ASD, unlike major gene mutations such as those in *Shank3* or *Fmr1*. Studies routinely compare the BTBR homozygote to a WT line, without studying the heterozygote phenotype. There is no widely available “BTBR *T+*” strain without *Itpr3^tf^* for direct comparison. The *tf* allele is essentially part of the BTBR package—you cannot currently order a “*tf*-negative” BTBR strain from a standard supplier [50,74].

Despite ignoring the *Itpr3* mutation as a target, over the past 20 years many interventions have been tested on BTBR mice to reverse or ameliorate its ASD-like behaviors. These interventions span pharmacological, dietary, microbiome, epigenetic, and environmental strategies.

Pharmacological interventions include mGluR5 antagonists such as MPEP, which reduces repetitive behaviors [77], and selective negative allosteric modulators like GRN-529, which reduces both repetitive behaviors and social deficits [78]. Dopamine D2 receptor modulation has also been recommended, based on research findings of increased D2R density in the striatum of BTBR mice. Therefore, agents like risperidone and aripiprazole are suggested to normalize stereotypic behavior [79]. GABAergic interventions include nanoformulated bumetanide, an NKCC1 ion cotransport inhibitor. When injected into the medial prefrontal cortex (mPFC), it reduces hyperexcitability and improves social interaction [80]. Even epigenetic and environmental approaches have been included, such as histone deacetylase (HDAC) inhibition with sodium phenylbutyrate, which reduces repetitive self-grooming behaviors and rescues social and cognitive deficits [81]; and environmental enrichment and neurostimulation, which are both shown to improve sociability and reduce repetitive behaviors in BTBR mice [82]. Furthermore, dietary interventions have also been explored, such as PUFA supplementation (omega-3 fatty acids), which had minimal impact [83], and a ketogenic diet, which, in contrast, has shown reproducible benefits in terms of reducing repetitive grooming, anxiety, and improving vocal communication—especially when applied to juvenile BTBR mice [84,85]. More recently, the microbiome has become an area of growing interest, with evidence that a butyrate-producing probiotic improves intestinal barrier function and reduces BTBR behavioral abnormalities [86].

The key point is that all these therapeutic manipulations target behavioral phenotypes, but none target Itpr3 directly. Most interventions act on synaptic plasticity, the excitation–inhibition balance, GABAergic function, or global epigenetic modulation. The unique mechanistic implications of the *Itpr3^tf^* allele—and the BTBR strain’s apparent ITPR dysfunction in tastebud signaling—have been left largely unexplored in therapeutic studies to date, except in terms of taste perception [69].

One mechanism by which the ITPR potentially contributes to ASD is via its control over cell death and survival decisions. This occurs through the regulation of ER calcium release and, consequently, autophagy, apoptosis, and impacts on cellular proliferation and migration. These processes are critical, not just in neurodevelopment but also in cancer, where ITPR3 has recently emerged as a key regulator [87,88,89].

## 3. Final Synthesis

Putting all this together, a powerful idea emerges, one saying that autism reflects a mismatch between the evolutionary demands of our modern brains and the metabolic systems that support them. The strongly supportive observations are as follows:

- The evolutionary importance of—and potentially critical historical, ecological- niche- permissive—newly-evolving energy-hungry enlarged human “social brain”, conveying the uniquely human set of “theory of mind” capabilities underlying language and culture, but that are specifically challenged in ASD.
- The critical role of ITPR channel gating in calcium signals that homeostatically control mitochondrial oxidative metabolic energy production, and the molecular ITPR gating defect that compromises calcium signaling and energetics that is observed in patients with an ADOS-confirmed diagnosis of ASD.
- The unique BTBR mouse model of ASD has cryptically harbored a unique missense mutation of that same mechanism, the *Itpr3* gene, which causes a syndrome not merely of taste dysfunction and unusual hair growth but also of *all* conventional ASD behavioral abnormalities, as well as an additional broad, recognizably patient-related set of physiological abnormalities of the gut, immune cells, and sensory systems, all of which are related to its ability to model a *typical ASD syndrome* and are already mechanistically linked to signaling via the ITPR.

This synthesis implies that our social, symbol-using, enlarged energy-hungry brains gave us language, art, weapons, and culture, allowing us to “read each other’s minds”, thereby to form conquering tribes that are larger than family units and “emerge from Africa” to cover the entire globe [90,91], but these brains also created a huge energetic burden. If something disrupts the delicate balance of energy supply during brain development, whether due to mitochondrial issues, calcium signaling problems, or environmental stressors, this may cause these newly evolved energy-demanding networks to fail and tip the scale toward developmental conditions like autism. This theory does not suggest that evolution “caused” autism. Instead, it offers a new framework for understanding why certain children may be more vulnerable, especially when their developing brains do not receive the necessary energy support that they need. It also opens new paths for intervention. If ITPR signaling and mitochondrial energy metabolism play a role in ASD, this might allow specific targeted diagnostics and therapeutics to be developed. Treatments that support mitochondrial function or improve calcium signaling could be beneficial for some individuals. With new specific targets come new interventions. Research in these areas is still evolving, but early findings are promising.

## 4. A Hopeful Future

ASD is complex and multifaceted. No single explanation will account for every individual’s experience. However, by looking back at our evolutionary past and connecting it to the molecular, cellular, and systems realities of the modern brain, we may understand the biological roots of autism and how to better support those on the spectrum today.

Understanding ASD through the lens of energy metabolism opens the door to novel diagnostics and interventions. One company at the forefront of this approach is NeuroQure (Neuroqure.com), which offers the *ASD Insight^®^* test. *ASD Insight^®^* is a first-of-its-kind offering that analyzes calcium-signaling abnormalities in patient-derived cells as early as two days after birth, through a discarded foreskin or minimally invasive skin sample [92]. By measuring how calcium flows through ITPR channels that are found in all cells of the body and that play a critical, decisive step in transducing a host of cellular signals into action, the test helps identify mitochondrial and cellular signaling patterns that may contribute to the development of ASD symptoms. This cellular molecular biophysical analysis represents a significant leap toward more biologically informed autism diagnostic solutions and treatments that support metabolic resilience in the brain.

It is important to note that when it comes to ASD, early detection is crucial. This allows for tailored interventions during critical developmental windows, enhancing communication, social skills, and overall quality of life for children with ASD [93,94,95]. By identifying potential challenges before behavioral symptoms emerge, families and healthcare providers can proactively plan and implement strategies that support optimal developmental outcomes.

In summary, the identification of the evolutionary role of mitochondrial energetics and synaptic signaling lipids in the emergence of our worldwide, large hyper-prosocial brain that led to the development of modern human-specific, culture-inducing prosocial skills, along with the variant or mis-expression of many of these late-evolving skills in the phenotype of autism, represents significant findings. In addition, the same battery of deficits being recognized in the best mouse model of ASD, which, by happenstance, has a unique mutation and a unique defect in function of the Itpr3 receptor channel, makes a compelling rationale for how an assay of this ITPR function, even in the skin cells from a newborn child, could provide a window into vulnerability to ASD. This discovery is above and beyond the empirical finding that an assay of this ITPR molecular dysfunction provides a 75–85% AUC ability to distinguish ASD [29]. Perhaps by turning our focus to this well-understood mechanism, rather than broadcast agnostic multi-omics hunts, we may, first, be able to hasten diagnosis, and therefore supportive therapy, but then perhaps next, an era of precision guided therapeutics to a known structure.

The structure of the inositol 1,4,5-trisphosphate receptor type 3 (ITPR3), encoded by the *ITPR3* gene, has been elucidated through advanced structural biology techniques. Notably, a high-resolution cryo-electron microscopy (cryo-EM) structure of the human ITPR3 was recently published, providing detailed insights into its architecture and functional mechanisms [96]. These structural studies demonstrate the structural heterogeneity of ITPRs in the presence of IP_3_, ATP, and Ca^2+^; correlating these structures with their plausible functional states should allow the definition of conformational changes at different gating states that underpin ITPR activation and gating. These structures could prove to be foundational for dissecting the function of the “*tf*” mutation and potential therapeutics targeted at the functionally implicated target for ASD therapy, as has been performed for other ion channels underlying other channelopathies [97,98,99,100,101].

## Abbreviations

ITPR	inositol 1,4,5-trisphosphate receptor
ER	endoplasmic reticulum
BTBR	a specific mouse strain developed in the 1950s
ROS	reactive oxygen species
HAR	human accelerated regions
PUFA	polyunsaturated fatty acids
FADS	fatty acid desaturase genes
DMN	default mode network
BBB	blood-brain barrier
SOCE	store-operated calcium channels
SERCA	sarcoplasmic/endoplasmic reticulum calcium ATPase
UPR	unfolded protein response
IRE1	inositol-requiring enzyme 1
XBP1	an endonuclease
ERAD	ER-associated degradation
PERK	protein kinase RNA-like ER kinase
eIF2α	α-subunit of eukaryotic initiation factor 2
ATF6	activating transcription factor 6
VDAC	mitochondrial voltage-dependent anion channel
GRP75	glucose-regulated protein 75
MCU	mitochondrial calcium uniporter
STIM1	stromal interaction molecule 1
MAM	mitochondrial-associated membrane
PDH	pyruvate dehydrogenase
IDH	isocitrate dehydrogenase
α-KGDH	α-ketoglutarate dehydrogenase
NADH	nicotinamide adenine dinucleotide (in its reduced form)
FADH_2_	flavin adenine dinucleotide (in its reduced form)
ETC	electron transport chain
P2Y	P2Y purinergic receptor
iPSC	induced pluripotent stem cells
FLIPR	fluorescent light plate reader
ROC	receiver operator characteristic
AUC	area under the curve
ADOS	Autism Diagnostic Observation Schedule
fMRI	functional magnetic resonance imaging
MRI	magnetic resonance imaging
EEG	electroencephalogram
NIRS	near-infrared spectroscopy
tf	tufted allele in mice
T	homozygous lethal allele at the brachyury locus
PKU	phenylketonuria
Abs	antibodies
MHC	major histocompatibility locus
F1	first filial offspring
Ig	immunoglobulin
FITC	fluorescein isothiocyanate
TNF-α	tumor necrosis factor alpha
IL-6	interleukin-6
BTBR T+ *Itpr3*^tf^/J	full name of the BTBR mouse strain from Jackson Labs
*Itpr3*^tf^	full name of the tufted allele in the BTBR mouse
BDNF	brain-derived neurotrophic factor
CRISPR/Cas9	clustered regularly interspaced short palindromic repeats/CRISPR-associated protein
mGluR5	metabotropic glutamate receptor 5
MPEP	antagonist of mGluR5
GRN-529	negative allosteric modulator of mGluR5
mPFC	medial prefrontal cortex
GABA	gamma-aminobutyric acid
